# A panel data study on the role of clean energy in promoting life expectancy

**DOI:** 10.1016/j.dialog.2024.100201

**Published:** 2024-12-20

**Authors:** Amit Roy

**Affiliations:** Department of Economics, Shahjalal University of Science & Technology, Sylhet-3114, Bangladesh

**Keywords:** Access to clean fuels and technologies, Life expectancy, Food deficit, Panel data

## Abstract

**Purpose:**

Energy is a health issue. Energy intersects with health outcomes, as evidenced by the relationship between access to clean fuels and technologies and population health measured by life expectancy at birth.

**Methods:**

Utilizing a comprehensive dataset spanning 190 countries from 2000 to 2022, this paper employs a range of static and dynamic panel data models to analyze this empirical relationship, while effectively managing unobserved country-specific heterogeneity and endogeneity issues.

**Results:**

The primary finding underscores that improved access to clean fuels and technologies positively correlates with increased life expectancy for both genders, males and females, on a global scale. Additionally, the study identifies a significant negative impact of food and nutritional deficiencies on human health, while highlighting positive associations between health outcomes and increased per capita health spending, immunization rates, education levels, and access to safe drinking water and sanitation facilities.

**Conclusion:**

Findings underscore the importance of policy interventions aimed at alleviating clean energy poverty and scaling up access to clean fuels and technologies to enhance both the duration and quality of life, thus fostering sustainable development efforts at both national and global levels.

## 1 Introduction and background

### 1.1 Why to promote life expectancy

Development encompasses multifaceted efforts aimed at enhancing both quantitative and qualitative aspects of human well-being. Central to this endeavor is the aspiration to improve the capability of individuals to lead long, healthy, and fulfilling lives [1]. Health stands as a cornerstone of development, influencing not only the duration but also the quality of life and livelihoods. Indeed, the development of health serves as a pivotal component of the broader development process, impacting various dimensions of human existence, including physical, social, and economic aspects. By investing in health, societies not only foster individual well-being but also lay the foundation for sustainable development, as healthy populations are better equipped to contribute productively to social and economic progress. Moreover, integrating health considerations into development strategies can yield synergistic benefits, as improvements in health outcomes can contribute to enhanced productivity, reduced poverty, and increased social cohesion. Ultimately, by placing health at the forefront of development agendas, policymakers can pave the way for more inclusive, sustainable, and equitable development pathways that prioritize the well-being of all individuals and communities.

The World Health Organization (WHO) has underscored the significance of life expectancy as a key indicator of national health status, surpassing traditional metrics such as mortality rates, disease prevalence, or disability rates [2]. This recognition reflects the comprehensive nature of life expectancy as a measure, encapsulating not only the presence or absence of disease but also the broader aspects of well-being and longevity [3]. As such, improving life expectancy has emerged as a paramount objective for both economic and public policymakers worldwide [4]. Recognizing the profound implications of longer life expectancy on societal well-being and economic productivity, policymakers are increasingly prioritizing interventions aimed at extending life expectancy and enhancing overall population health. In alignment with the global focus on improving health outcomes, the Sustainable Development Goals (SDGs) introduced by the United Nations represent a groundbreaking initiative aimed at advancing the well-being of present and future generations. Among the myriad of targets outlined in the SDGs, increasing global average life expectancy stands out as a central objective. By setting a target to increase global average life expectancy at birth by 4 years by 2030, the SDGs signal a collective commitment to addressing disparities in health outcomes and promoting equitable access to healthcare services worldwide [5]. This ambitious target underscores the recognition of life expectancy as a fundamental determinant of human development and underscores the urgency of concerted efforts to improve health outcomes on a global scale.

### 1.2 Energy as a health issue

Energy is a health issue [6]. The intersection of energy and health underscores a fundamental aspect of human well-being and societal development. Energy access, or the lack thereof, significantly influences various dimensions of public health, ranging from basic necessities such as cooking and heating to healthcare delivery and disease prevention. In many parts of the world, particularly in low- and middle-income countries, reliance on inefficient and fossil energy sources, such as solid fuels and biomass, contributes to indoor air pollution, respiratory diseases, and premature mortality [7]. Latest statistics reveal that more than three billion people, 39 % of the global population, rely on solid and fossil fuels like biomass, charcoal, coal, kerosene, crop residues, dung, or wood to meet their primary energy needs for cooking [8]. The smoke arising in the household from the combustion of such fuels is laced with health-damaging pollutants like carbon monoxide, nitrogen dioxides, and poly-aromatic hydrocarbons [9]. Penetrating deep into the body of its victims, this smoke causes a range of deadly chronic and acute health effects such as pneumonia, lung cancer, chronic obstructive pulmonary disease, and heart disease as well as low birth weights in children born to mothers whose pregnancies are spent breathing toxic fumes from open fires of stoves [10]. It is now the 5th biggest killer of the globe exceeding deaths attributable to malaria or tuberculosis [11]. The *kitchen killer* turned out to be responsible for 4.3 million deaths per year globally including nearly 12of pneumonia deaths in children under age 5; 13of deaths from chronic obstructive pulmonary disease; nearly 15of deaths of ischemic heart disease; and more than 5 % of the global burden of disease in 2020 where 60 % of victims were women [12]. Moreover, indoor air pollution is responsible for more deaths than road accidents (1·4 million) as well as responsible for 3 times as many deaths as AIDS, tuberculosis, and malaria combined and for nearly 15 times as many deaths as war and all forms of violence [13].

On the other hand, access to modern energy systems impacts human well-being by reducing health risks associated with traditional energy use [14]. Transitioning from traditional fuels, such as solid biomass and kerosene, to cleaner and more efficient energy sources can significantly reduce indoor air pollution and related health problems. By expanding access to modern energy systems, such as clean cookstoves, solar lighting, and electric heating, communities can substantially improve indoor air quality and reduce the burden of preventable diseases, thus enhancing overall health and well-being. Additionally, increasing the use of clean fuel and technologies brings about the greatest co-benefits of health and climate protection [15]. Clean energy solutions, such as renewable energy sources (e.g., solar, wind, and hydroelectric power) and energy-efficient technologies, not only reduce greenhouse gas emissions and mitigate climate change but also yield significant health benefits. By minimizing air pollution, promoting sustainable transportation, and enhancing energy efficiency, clean energy initiatives contribute to improved public health outcomes, including reduced rates of respiratory illnesses, cardiovascular diseases, and premature mortality. Therefore, investing in clean energy infrastructure and technologies represents a critical pathway towards achieving synergistic gains in both health promotion and climate resilience, ultimately fostering a healthier and more sustainable future for all.

### 1.3 Life expectancy and clean energy trends and trajectories

**Fig. 1:**
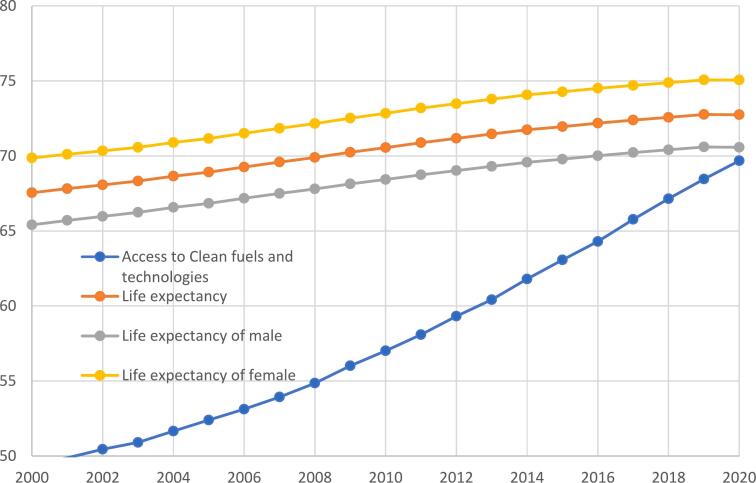
**Global Trends of Life Expectancy and Clean Energy.** The figure is based on World Bank [16]. Life expectancy at birth total, male and female is in years and access to clean fuels and technologies (% of the population) are scaled in the vertical axis.

**Fig. 2:**
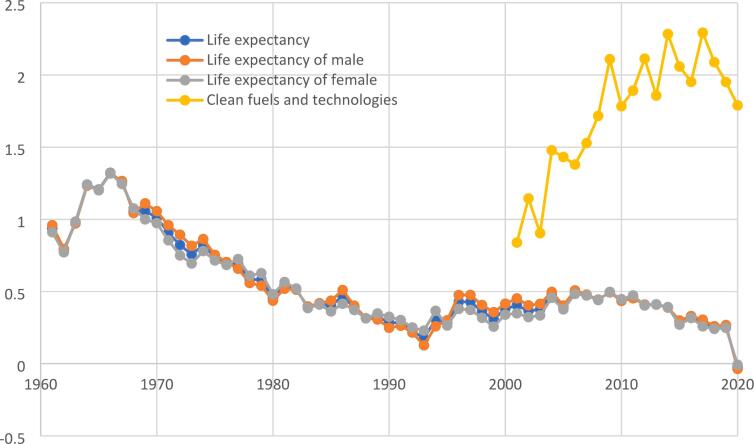
**Growth Rates of Life Expectancy and Clean Energy.** The figure is based on World Bank [16]. The rate of growth of life expectancy at birth and access to clean fuels and technologies are scaled in the vertical axis. Data on Access to clean fuels and technologies before 2000 is not available.

World Bank [16] data reveals the unprecedented progress of life expectancy at birth and access to clean fuels and technologies during the first one and half decades of the twenty-first century (Fig. 1). There was a global increase of 2.5 years in life expectancy between 2000 and 2020. Global average life expectancy at birth in 2022 reaches 72.7 years whereas only 29 countries have an average life expectancy of 80 years or higher. On average, women live longer than men in every country of the world wherein female life expectancy at birth is 75 years and male life expectancy at birth is 71 years on average. Nevertheless, Fig. 2 portrays that the rate of improvement of female life expectancy at birth is much lower than the rate of improvement of male health since 1995.

Moreover, it is visible from Fig. 2 that despite some notable progress, the rate of improvements towards cleaner energy is becoming slower which eventually decreases the rate of improvements in life expectancy momentously. The fall in the growth of clean fuel and technologies since 2005 is likely attributed to the falling prices of fossil fuel energies like oil, innovation of shale gas, increasing cost in the promotion of green technologies as well as the demand for least cost fuels in the developing nations [17].

It is worthwhile to notice here that the falling rate of the improvement in life expectancy followed the same cycle of the falling rate of progress in access to clean fuels and technologies. Although the proportion of the global population with access to clean fuels and technologies for cooking has reached full coverage in Europe (>95 %) and the Americas (>92 %), only 17 % of the population has access to clean fuels in the African Region. Table 1 compares access to clean fuels and technologies with the highest life expectancy against the bottom ten countries with the lowest life expectancy. It reveals that countries with less percentage of the population having access to clean fuels and technologies are at the bottom of the life expectancy table. Strikingly, the bottom countries in the LE table are from the African region where most of the countries have extremely low access to clean energy.

**Table 1:** Life expectancy at birth (LE) and Access to clean cooking energy (A2C).

Top 10 Countries in terms of LE			Bottom 10 Countries in terms of LE		
Country	LE	A2C	Country	LE	A2C
Hong Kong	85.4	100 %	Central African Republic	53.7	0.8 %
Japan	84.6	100 %	Chad	54.5	6.8 %
Macao, China	84.3	100 %	Lesotho	54.8	40.1 %
Singapore	83.7	100 %	Nigeria	55.0	15 %
South Korea	83.4	100 %	Sierra Leone	55.1	0.8 %
Norway	83.2	100 %	Somalia	57.7	3.2 %
Australia	83.2	100 %	South Sudan	58.1	0.0 %
Switzerland	83.1	100 %	Cote d'Ivoire	58.1	31.8 %
Iceland	83.1	100 %	Africa Western Central	58.4	14.9 %
Israel	82.7	100 %	Guinea-Bissau	58.6	1.1 %

Source: World Bank [15]. Life expectancy (LE) at birth is reported in years and access to clean fuels and technologies (A2C) is reported as a percentage of the total population of the country.

Against this backdrop, this study endeavors to explore the interplay between access to clean fuel and technologies and the increase in life expectancy on a global scale. While a plethora of research exists on the determinants influencing life expectancy, notably absent is an investigation into the empirical relationship between access to clean fuel and technologies and life expectancy at birth. This study seeks to bridge this gap in the literature by examining this association comprehensively. The paper is structured as follows: the subsequent section provides an overview of existing literature on factors affecting life expectancy and establishes the conceptual framework for the study. Section 3 and its subsections detail the empirical methods and variables employed in the analysis. Section 4 elucidates the results and key findings. Finally, section 5 concludes the study by proposing policy recommendations.

## 2 Literature review and conceptual framework

### 2.1 Determinants of life expectancy in the existing literature

The first and foremost determinant of population health in the existing literature is percapita real income [18]. However, an increase in income does not automatically translate into higher life expectancy [19], rather its significance lies in the way that the benefits of higher income are publicly distributed protecting and effectively promoting the entitlements of people [20]. The most obvious explanation is that higher income percapita ensures food security, access to education, health care, epidemiological protection, housing, and other amenities which lead to improved health and higher life expectancy [21,22]. Similarly, access to safe drinking water [23], sanitation [24], better nutrition [25], and literacy [26] are crucial determinants of life expectancy rather than income per capita. Merely having higher income does not guarantee higher life expectancy; rather, the significance lies in how the benefits of increased income are distributed within society. Effective public distribution mechanisms play a crucial role in ensuring that the advantages of higher income are equitably shared among individuals, thereby safeguarding and promoting the entitlements of all members of the population. Therefore, while income plays a vital role in shaping health outcomes, its impact is contingent upon the implementation of policies and strategies that prioritize equitable distribution and access to resources and services necessary for maintaining and enhancing population health.

The findings from Bloom et al. [27] underscore the pivotal role of immunization as a cost-effective tool for improving population health, particularly in combating major diseases since World War II. Immunization programs have been instrumental in preventing the spread of infectious diseases and reducing morbidity and mortality rates worldwide. By effectively immunizing populations against prevalent diseases such as measles, polio, and influenza, public health initiatives have achieved significant gains in life expectancy and overall well-being. These findings carry profound implications for public health policy, highlighting the importance of sustained investment in immunization programs as a cornerstone of disease prevention and control efforts. Moreover, recognizing the cost-effectiveness of immunization underscores the need for continued support and funding for vaccination campaigns, particularly in resource-constrained settings where access to healthcare services may be limited.

Moreover, the research by Monsef and Mehrjardi [28] sheds light on the social and economic determinants of life expectancy, revealing the adverse impact of unemployment and inflation on population health outcomes. Unemployment not only leads to economic insecurity and reduced access to healthcare services but also contributes to psychosocial stressors, which can exacerbate health inequalities and undermine well-being. Similarly, inflationary pressures can erode purchasing power and affordability of essential goods and services, including healthcare, thereby impeding individuals' ability to maintain their health and well-being. These findings have important implications for economic and social policy, emphasizing the need for measures aimed at promoting stable employment opportunities and controlling inflation rates to safeguard population health and enhance life expectancy. Addressing structural factors such as unemployment and inflation can contribute to more equitable and resilient societies, ultimately fostering better health outcomes and overall societal well-being.

### 2.2 Theoretical links between household use of clean energy and life expectancy

Household clean cooking energy use profoundly impacts life expectancy through the reduction of indoor air pollution. In many low and middle-income countries, households rely on traditional biomass for cooking exposing household members to death by respiratory, pulmonary, and cardiovascular diseases [29]. On the other hand, clean household energy reduces indoor air pollution by replacing traditional and solid fuels, such as firewood, coal, charcoal, and dung, etc., which emit harmful pollutants like particulate matter carbon monoxide, and volatile organic compounds during combustion. These pollutants contribute to a range of respiratory and cardiovascular diseases, particularly in households that rely on inefficient cooking methods in poorly ventilated spaces. Clean energy sources, such as electricity, solar, biogas, and liquefied petroleum gas (LPG), produce little to no harmful emissions, drastically lowering the concentration of indoor pollutants. By providing a cleaner, more efficient energy source, clean energy improves indoor air quality, reducing the health risks associated with long-term exposure to toxic fumes, and thereby enhancing overall health and life expectancy.

Beyond the immediate reduction of health risks, access to clean cooking energy also influences life expectancy through socioeconomic channels. Traditional cooking methods often require long hours spent gathering fuel, a task that disproportionately falls on women and children, particularly in rural areas [30]. This labor-intensive activity exposes them to physical risks and limits their time for education, income-generating activities, and social participation. Clean cooking solutions reduce the need for fuel collection, freeing up time for more productive activities, which can lead to better economic outcomes, improved food security, and higher standards of living [31].

Furthermore, the impact of clean energy access extends to nutritional outcomes, which also influence life expectancy. Households using clean energy can prepare food more safely and efficiently, reducing the risk of foodborne illnesses associated with traditional cooking methods [32]. Improved nutrition, in turn, leads to better health outcomes, as adequate access to diverse and safe foods is crucial for immune function and overall vitality [33].

Additionally, climate change negatively affects life expectancy by rising temperatures and altered rainfall patterns [34]. It increases the frequency and intensity of extreme weather events, natural disasters, and the spread of vector-borne diseases like malaria and dengue, which ultimately kill millions of people. Clean energy mitigates these impacts by reducing greenhouse gas emissions, thereby reducing the incidence of climate-related health risks and contributing to longer and healthier lives. This comprehensive connection between clean energy, reduced health risks improved nutrition, and climate security underscores the critical role of clean energy in promoting health and longevity.

### 2.3 Conceptual framework of the study

**Fig. 3:**
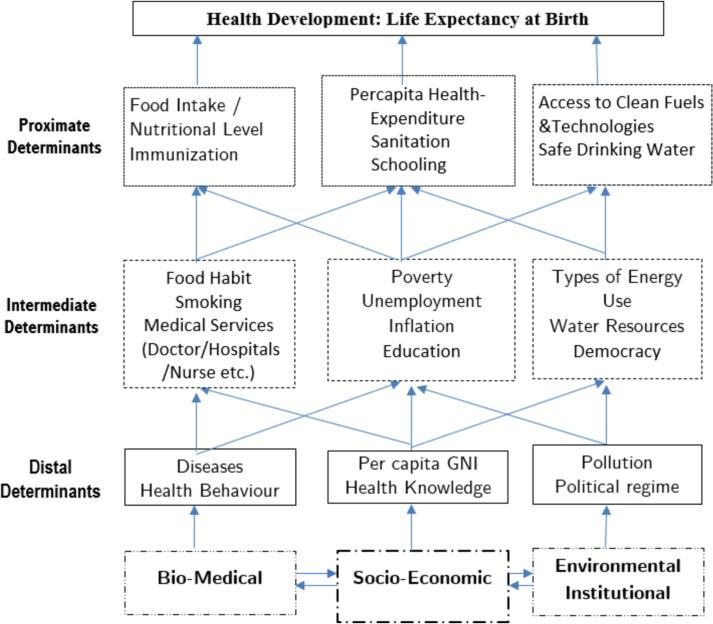
**Determinants of Life Expectancy.** The author's build-up is based on Barlow and Vissandjée [35] and Schell et al. [36].

The conceptual framework proposed by Barlow and Vissandjée [35], expanded upon by Schell et al. [36], offers a comprehensive model for understanding the determinants of life expectancy. Based on this formulation, we sketch the conceptual framework for this study (Fig. 3). This model distinguishes between proximate, intermediate, and distal determinants, highlighting the direct and indirect effects of various factors on life expectancy at birth. Proximate determinants are those variables that directly affect life expectancy and are exogenous to the dependent variable. In contrast, immediate and distal determinants include factors that exert both direct and indirect influences on life expectancy, operating through proximate determinants. By delineating the complex pathways through which different determinants interact and influence longevity, this framework provides a valuable tool for guiding empirical research and policy interventions aimed at improving life expectancy and overall well-being. Drawing upon this conceptual framework, the present study aims to explore the synergy between access to clean fuel and technologies and gains in life expectancy at the cross-country level.

Summing up the 2.1, 2.2 studies, we can infer that, the determinants of life expectancy are multifaceted and can be broadly categorized into socio-economic, medical-biological, and environmental-institutional factors. Socio-economic catalysts such as income per capita, per capita health expenditure, education, and access to public goods like sanitation play a significant role in shaping population health outcomes. Medical-biological catalysts encompass variables related to food intake, nutrition, diseases, immunization, and pharmaceutical consumption, which directly impact health status and disease prevalence within populations. Finally, environmental-institutional catalysts, such as the type of energy use, availability of clean drinking water, and political regime, exert both direct and indirect effects on life expectancy by shaping environmental conditions, healthcare infrastructure, and governance frameworks. By integrating insights from socio-economic, medical-biological, and environmental-institutional determinants, the study seeks to elucidate the multifaceted nature of the relationship between clean energy access and population health outcomes. By employing rigorous empirical methods and robust statistical models, the study aims to provide evidence-based insights into the potential pathways through which clean energy access influences life expectancy, thus informing policy decisions and interventions aimed at promoting public health and sustainable development. The findings from this study are expected to have important implications for public health policy and sustainable development initiatives.

## 3 Empirical method and data

### 3.1 Econometric method

To investigate the cross-national relationship between life expectancy and economic, bio-medical, and environmental factors, the panel data method is used in this study. Panel data models are chosen for their ability to capture both cross-sectional and time-series variations, allowing us to control for unobserved heterogeneity that might affect the relationship between clean energy access and life expectancy. By using panel data, we can account for individual differences across countries or regions that remain constant over time, as well as capture dynamic changes within units over the study period. This makes panel data particularly suitable for analyzing the long-term effects of clean energy on life expectancy, as it controls for country-specific factors and temporal trends. While challenges such as potential multicollinearity or endogeneity issues were encountered, these were addressed by employing appropriate techniques, such as fixed effects or random effects models, and conducting robustness checks. This ensures the reliability and validity of the findings, making panel data an appropriate and robust methodological choice for this study.

#### 3.1.1 Pooled OLS

Pooled Ordinary Least Squares (OLS) is the first-panel data estimator which assumes that the intercepts are homogeneous, namely $\alpha_{i}=\alpha$, for all i. Given a panel sample of N countries over T periods, the basic linear regression equation takes the form of Pooled OLS as:

$${\mathrm{LE}}_{\mathrm{it}}=\alpha +X_{\mathrm{it}}^{'}\beta +e_{\mathrm{it}}\forall i=1,\ldots ,N;t=1,\ldots ,T$$

Here, LE stands for life expectancy, the dependent variable of the study. $X_{\mathrm{it}}$ is the vector of explanatory variables where

$$X_{\mathrm{it}}=\left[\begin{array}{c} \text{Access to Clean Fuels and Technologies}\;\left(A2C\right)\\ \text{Food Deficit}\;\left(\text{Kcals}\;\mathrm{per}\;\text{person}/\mathrm{day}\right)\;\text{or Undernourishment}\;\\ \text{Health Expenditure}\;\mathrm{Per}\;\text{Capita}\\ \text{Primary Education}\\ \text{Immunization}\\ \text{Safe Drinking Water}\;\\ \text{Improved Sanitation} \end{array}\right]$$

α denotes accounts for any individual country specific effect that is not included in the regression and $e_{\mathrm{it}}$ is the error term, i. I. d.$∼\left(0,\sigma^{2}\right)$. α and β can be estimated. It is worthwhile to mention here that in addition to working through proximate determinants like food, nutrition etc., the distal determinant, percapita real income shares linear dependence with health expenditure percapita which creates multicollinearity problems [37]. In order to avoid such inaccuracy in the estimation, this paper has dropped percapita income from the list of explanatory variables.

#### 3.1.2 Fixed effect model

Fixed effect model rewrites pooled OLS model by introducing an unobservable country specific time-invariant effect,$\;u_{i}.$ If individual effect $u_{i}$ (cross-sectional or time specific effect) does not exist ($u_{i}$=0), pooled OLS produces efficient and consistent parameter estimates. Symbolically,

${\mathrm{LE}}_{\mathrm{it}}=\alpha_{i}+X_{\mathrm{it}}^{\prime }\beta +e_{\mathrm{it}}$ where $\alpha_{i}=\alpha +u_{i.}$

However, when individual effects are not zero ($u_{i}\neq 0)\;$in longitudinal data, heterogeneity (individual specific characteristics) yields biased estimators, that is, pooled OLS estimator is no longer the best unbiased linear estimator (BLUE). Fixed effect model models provide a way to deal with these problems.

#### 3.1.3 Random effect model

Panel data models examine group (individual-specific) effects, time effects, or both in order to deal with heterogeneity or individual effect that may or may not be observed. These effects are either fixed or random effect. Whereas a fixed effect model examines if intercepts vary across groups or time periods, a random effect model explores differences in error variance components across individuals or time periods:

$${\mathrm{LE}}_{\mathrm{it}}=\alpha +X_{\mathrm{it}}^{'}\beta +\epsilon_{\mathrm{it}}\;\mathrm{where}\;\epsilon_{\mathrm{it}}=u_{i}+e_{\mathrm{it}}$$

Here the random effect is included into $u_{i}.$

The validity of fixed effects is tested by the F-test, while the presence of random effects is examined by the Lagrange multiplier (LM) $\chi^{2}\;$-test [38]. If the null hypothesis is not rejected in either test, the pooled OLS regression is favored. However, if the null hypothesis is rejected in both tests, we are used to being in dilemma in choosing the appropriate model. Hausman [39] devised a specification test based on the difference between the fixed and random effects estimators. The Hausman specification test examines if the individual effects are uncorrelated with other regressors in the model. If individual effects are correlated with any other regressor, the random effect model violates a Gauss-Markov assumption and is no longer the best linear unbiased estimator [40]. Therefore, if the null hypothesis is rejected, a fixed effect model is favored over the random counterpart. If the null hypothesis is not rejected, we can conclude that there is a significant random effect in the panel data.

#### 3.1.4 Panel System and Difference GMM

The Generalized method of moments (GMM) is generally used to address temporal and endogeneity issues that arise from reverse causality and omitted variable bias in panel data studies [41]. Unlike static models such as pooled OLS, fixed effect, or random effect, GMM allows for the inclusion of lagged dependent variables, capturing the dynamic nature of the relationship. This dynamic aspect is essential when considering how past levels of life expectancy may influence current outcomes. Additionally, GMM accounts for the potential correlation between the independent variables and the error term, offering more reliable and efficient estimates in the presence of such endogeneity.

The least-square estimators, i.e., fixed effect or random effect estimators are inconsistent for a finite number of time periods and a large number of cross-section observations. The Arellano and Bond [42]’s *Difference GMM* estimator is designed for datasets with many cross-sections and few periods. Difference GMM transforms the model into first differences - to wipe out the individual-specific effects. Sequential moment conditions are then used where lagged levels of the variables are instruments for the endogenous differences. In contrast, Arellano and Bover [43], Blundell and Bond [44]’s *system GMM* propose the use of extra moment conditions arising from the model in levels when certain stationarity conditions of the initial observation are satisfied. The resulting System GMM estimator combines moment conditions for the model in first differences with moment conditions for the model in levels. Here,

$${\mathrm{LE}}_{\mathrm{it}}=\sum_{j=1}^{\rho }\alpha_{j}{\mathrm{LE}}_{i,t-j}+X_{\mathrm{it}}^{\prime }\beta +u_{i}+e_{\mathrm{it}}$$

Where p =1, 2, …j are the lag associated with endogenous covariates. Both Difference and System GMM estimators use instruments which are available from within the system of equations as no external instruments are available to us.

In this study, the conceptual framework visualizes potential endogeneity in life expectancy determination because causality may run in both directions – from health to education and vice versa and these regressors may be correlated with the error term. Moreover, the life expectancy in the past period is also a potential determinant of future life expectancy [45] implying that the lag of life expectancy is also an explanatory variable in dynamic stance. System and Difference GMM are the best available econometrics methods to deal with lags and endogeneity in dynamic panel data settings. Therefore, we use them in our study.

### 3.2 Data and variables description

This paper employs the World Bank [16] data to empirically investigate the role of clean energy in promoting life expectancy. Life expectancy at birth is used as the dependent variable. Moreover, the life expectancy of males at birth and the life expectancy of females at birth are used in sensitivity analysis. The data on life expectancy at birth (LE), life expectancy of males at birth (MLE), and life expectancy of females at birth (FLE) are available from 1960 to 2022 for 217 countries. The main explanatory variable of the paper is access to clean fuels and technologies (A2C). A2C is defined as the proportion of the total population primarily using clean fuels and technologies in households for cooking, heating, and lighting purposes. The data on A2C is available only from 2000 to 2020 for 190 countries, thus, this study is limited to the timespan.

To control the effect of nutritional deficiency on life expectancy, two alternative indicators—depth of the food deficit (kilocalories per person per day) and undernourishment are applied. The depth of the food deficit indicates how many calories would be needed to lift the undernourished from their status. On the other hand, undernourishment is defined as the prevalence of malnutrition among the percentage of children under age five. To control the effect of distal parameter income on health, we use health expenditure percapita as an indicator. It is the estimate of current health expenditures during each year expressed in international dollars at purchasing power parity (PPP). Moreover, as a proxy of disease control instrument, we employ immunization which is calculated as the percentage of children ages 12–23 months who received the measles vaccination before 12 months or at any time before the survey. In addition, to examine the effect of consciousness and health knowledge on life expectancy, we use primary education as a proxy variable. Primary education is measured as the percentage of the total population who passed the last grade of primary class regardless of age.

To control the effect of public health facilities, we incorporate access to safe drinking water and sanitation. Safely drinking water is computed as the percentage of the population with reasonable access to an improved water source, such as, a household connection, public standpipe, borehole, protected well or spring and rainwater collection on premises, available when needed, and free from fecal and priority chemical contamination. Improved sanitation facilities are defined as improved facilities, e.g., flush toilets connected to sewers not shared with other households.

## 4 Results and discussion

### 4.1 Statistical validity

Table 2 reports the results of our basic models. The first column inputs the list of the variables under study. The second column reports the pooled OLS result. The value of the F-statistic of pooled OLS tells us that the model is statistically valid at <0.001 level, however, the goodness of fit of the model is 0.796 measured by R^2^. To improve the goodness of the model and to control the country-specific heterogeneity, the fixed effect (FE) model is then estimated. FE model is also found statistically valid with <0.001 level of significance. Then we apply the random effect (RE) model which is also found statistically valid with <0.001 level of significance. We hereafter employ the Breusch-Pagan LM χ2 test to check the presence of random effect in the model. The null hypothesis of the LM χ2 test is that there is no random effect in the model. The estimated output rejects the null hypothesis at <0.01 level of significance. As both FE and RE models are found statistically valid, then we use the Hausman test to choose between these two models. The result of the Hausman test favors the fixed effect model as it rejects the null hypothesis at <0.001 level of significance.

**Table 2:** Determinates of Life Expectancy.

Life Expectancy at Birth (LE) is the dependent variable						
Model Variable	Pooled OLS	Fixed Effect	Random Effect		System GMM	Difference GMM
Access to Clean Fuels and Technologies (A2C)	0.028*** (0.002)	0.010*** (0.003)	0.012*** (0.004)		0.006*** (0.000)	0.007*** (0.001)
Food Deficit (Kcals per person/day)	−0.012*** (0.002)	−0.009*** (0.001)	−0.008*** (0.003)		−0.009*** (0.004)	−0.009*** (0.003)
Health Expenditure Per Capita	0.003*** (0.001)	0.007*** (0.001)	0.006*** (0.001)		0.001*** (0.000)	0.001*** (0.000)
Primary Education	0.002** (0.001)	0.064*** (0.005)	0.028*** (0.004)		0.002*** (0.000)	0.003*** (0.001)
Immunization	0.077*** (0.009)	0.031*** (0.009)	0.045*** (0.007)		0.006*** (0.001)	0.002*** (0.000)
Safe Drinking Water	0.059*** (0.012)	0.214*** (0.014)	0.226*** (0.019)		0.003** (0.001)	0.001** (0.000)
Improved Sanitation	0.071*** (0.005)	0.054*** (0.008)	0.070*** (0.011)		0.029*** (0.003)	0.011*** (0.003)
Life Expectancy_t-1_	–	–	–		0.981*** (0.002)	0.926*** (0.003)
Intercept	3.230*** (0.054)	1.970*** (0.069)	2.291*** (0.071)		0.137*** (0.009)	0.188*** (0.011)
N	1446	1446	1446		1349	1172
F or **χ**^**2**^ Statistic [p values]	704.970*** [0.000]	968.95*** [0.000]	1814.73*** [0.000]		1870000*** [0.000]	282,435.17*** [0.000]
R^2^	0.796	0.974				
ρ (rho)			0.930			
F test (Fixed Effect) [p values]		157.826*** [0.000]				
Breusch-Pagan LM χ^2^ test [p values]			6835.41*** [0.000]			
Hausman Test [p values]		343.96*** [0.000]				
AR (1) [p values]					0.002	0.002
AR (2) [p values]					0.101	0.100
Sargan [p values]					0.844	0.794

N.B.: ***, ** and * denotes significance of coefficients at level < 0.01, <0.05 and < 0.10 respectively.

() reports standard errors and [] reports *p* value.

### 4.2 Clean energy secures longevity

Irrespective of the Pooled OLS, FE, and RE model specifications, we find that access to clean fuels and technologies has a positive effect on the life expectancy at birth at <0.01 level of significance. For example, in the case of the FE model, the coefficient value of A2C is 0.010, which implies that the doubling the use of clean fuel and technologies will increase the life expectancy of birth by 1 % holding all other variables constant. To interpret, for instance, Bangladesh had a 72.43-year life expectancy at birth in 2019, if Bangladesh can double the use of clean fuel and technologies by 100 %, the life expectancy of birth will rise by 0.7243 years or almost 265 days, so the life expectancy would be 73.15 years then and so on. The findings suggest that increasing access to clean fuel and technologies can have significant positive effects on life expectancy at birth. For policymakers, this underscores the importance of investing in initiatives aimed at promoting the adoption of clean energy solutions, particularly in regions where access to modern energy sources is limited. By prioritizing clean energy infrastructure and technologies, policymakers can not only improve population health outcomes but also advance broader development goals, including poverty reduction, environmental sustainability, and economic growth.

Moreover, the observed relationship between clean energy access and life expectancy highlights the potential for targeted interventions to yield substantial gains in health outcomes, particularly in low- and middle-income countries where the burden of disease associated with traditional energy use is most pronounced. It has important policy implications for health equity and social justice too. Clean fuel and technologies is often unequally distributed, with marginalized and vulnerable populations disproportionately affected by the health risks associated with traditional energy sources. Addressing disparities in clean energy access is therefore essential for promoting health equity and ensuring that all individuals have the opportunity to live longer and healthier lives. Policymakers must prioritize interventions aimed at expanding clean energy access in underserved communities, while also addressing underlying social determinants of health, such as poverty, education, and access to healthcare. By adopting a comprehensive and inclusive approach to clean energy promotion, policymakers can advance health equity objectives, reduce disparities in life expectancy, and build more resilient and equitable societies for future generations.

Moreover, the significance of the intercept term in the FE model, observed at a level of <0.01, indicates the presence of substantial unobserved heterogeneity across the countries included in the analysis. This finding underscores the importance of accounting for country-specific characteristics and differences that may influence life expectancy outcomes beyond the variables explicitly included in the model. By capturing the effects of unobserved factors that are constant over time within each country, the intercept term provides valuable insights into the underlying determinants of life expectancy and highlights the need for robust statistical methods to control for such heterogeneity. Therefore, policymakers and researchers should carefully consider and address the complexities of unobserved heterogeneity when interpreting the results and formulating policy interventions aimed at improving population health outcomes.

To control the endogeneity, we afterward put GMM estimators into operation. The **χ**^**2**^ statistics confirm the overall significance of both the System and Difference GMM models at <0.001 level of significance. We then conduct the Sargen test which has the null hypothesis that overidentifying restrictions are valid. Since the result of the Sargen test fails to reject the null hypothesis at the conventional level of significance, the models are statistically valid. Moreover, we run AR (1) and AR (2) tests with the null hypothesis of no autocorrelation (AR). The AR (1) result presents strong evidence against the null hypothesis of zero autocorrelation in the first-differenced errors at order 1 which confirms the validity of the GMM models. Moreover, *p* values of AR (2) tests do not reject the null hypothesis, that is, there may not be any second order of serial correlation, hence only the first lag of the dependent variable is valid.

**Fig. 4:**
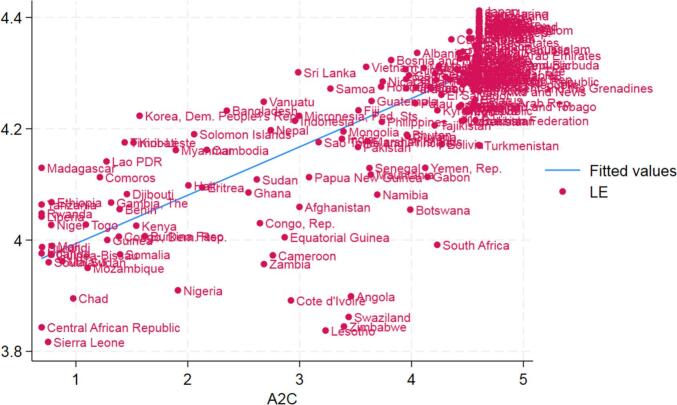
**Fitted Values of Log of Life Expectancy (LE) and Log of Access to Clean Fuels and Technologies(A2C).** The figure is drawn in Stata 18 as the output of post-estimation fitted values.

Similar to the static panel data models of POLS, FE, and RE models, dynamic panel data models, the System GMM, and Different GMM, reveal that access to clean fuels and technologies, per capita health expenditure, primary education, immunization, safe drinking water, and improved sanitation facilities have a positive effect on the life expectancy while nutritional deficiency has a negative effect on the life expectancy at birth at the conventional level of significance. From System GMM estimation, we find that access to clean fuels and technologies (A2C) is a significant determinant of life expectancy at birth with an elasticity of 0.006 at <0.01 level of significance. It tells us that if the percentages of clean fuels and technologies used in countries were doubled, average life expectancy for the population would increase by 0.6 %, remaining all other things constant. For instance, if the average life expectancy for the population of a country is 70 years, then doubling access to clean fuels and technologies would increase average life expectancy by 153 days, that is, by almost 5 months after passing the threshold age. The positive relationship between average life expectancy (LE) and access to clean fuels and technologies (A2C) is plotted in Fig. 4.

### 4.3 Effect of nutritional deficiency, education, and health facilities on life expectancy

In addition, we find that per capita health expenditure, primary education, immunization, safe drinking water, and improved sanitation facilities have a positive effect on the life expectancy at birth while nutritional deficiency measured by food deficit has a negative effect on the life expectancy at <0.01 level of significance. If the food deficit decreased by 10 %, average life expectancy would rise approximately by 229 days, that is, almost seven and half months, and so on. The observed correlation underscores the critical role of adequate nutrition in shaping population health outcomes and highlights the benefits of addressing food insecurity and malnutrition. For policymakers, this implies that interventions aimed at improving food security, promoting healthy diets, and addressing nutritional deficiencies can have significant positive impacts on life expectancy and overall well-being. Therefore, efforts to reduce food deficits and ensure access to nutritious food should be prioritized as part of comprehensive strategies for improving population health and advancing global development goals.

The finding that per capita health expenditure, primary education, immunization, safe drinking water, and improved sanitation facilities positively affect life expectancy at birth has significant policy implications. Governments should prioritize investments in these areas as essential components of public health infrastructure. Increased health expenditure can enhance access to medical services and improve overall healthcare quality. Expanding primary education contributes to greater health literacy and healthier behaviors. Immunization programs reduce preventable diseases while ensuring access to safe drinking water and improved sanitation can prevent waterborne diseases and improve overall living conditions. Policymakers should adopt a holistic, integrated approach, targeting these key determinants to improve population health outcomes and promote sustainable development.

### 4.4 Dynamics of life expectancy and why do we care

Moreover, the lag of the life expectancy at birth is also found a statistically significant determinant in the dynamic panel setting. The influence of past life expectancy on current outcomes highlights the importance of historical trends in shaping present health outcomes. It suggests that previous investments in health infrastructure, clean energy access, and other socio-economic factors have long-lasting effects. Historical trends in life expectancy interact with variables like clean energy access and nutritional deficits in that improvements in these areas may have cumulative and delayed effects on population health. For instance, access to clean energy can improve living conditions, reduce air pollution, and support better healthcare, which gradually contributes to higher life expectancy. Therefore, addressing the factors underlying temporal changes in life expectancy requires long-term and sustained efforts, as improvements or declines in life expectancy may reflect the cumulative effects of past policies, socioeconomic conditions, and public health interventions. As such, long-term policies should focus on creating an environment where these variables continue to evolve positively, with a focus on both immediate and future impacts.

Access to clean energy is not only about reducing carbon emissions and mitigating climate change, it is also about improving public health, particularly in developing regions where traditional energy sources often contribute to premature deaths. Furthermore, the question of “Why do we care?” extends to the long-term sustainability of public health. Investing in clean energy today means not only addressing current health challenges but also creating a healthier future. The positive effects of clean energy access on public health accumulate over time, with improvements seen in areas such as reduced air pollution-related illnesses, better maternal and child health, and a decrease in the number of preventable diseases. By recognizing the temporal lag between health outcomes, we are better equipped to design policies that ensure sustained progress in both the energy and health sectors. Long-term policy planning that integrates clean energy with health interventions is essential to break the cycle of poverty, improve living conditions, and promote equitable development, ultimately leading to healthier and more resilient populations for generations to come. Additionally, understanding the dynamics of life expectancy can inform targeted interventions aimed at addressing disparities and inequalities in health outcomes across different population groups and regions. By addressing the root causes of health disparities and promoting equitable access to healthcare services and resources, policymakers can work towards achieving more inclusive and sustainable health outcomes for all.

### 4.5 Sensitivity testing

Econometrics deals with complex multivariate relationships that are influenced by many factors. According to Leamer [46], when an inference is tested based on certain assumptions or data, sensitivity analysis is required to rebut doubt about the inferences. In modern empirical economic literature, estimation and sensitivity analysis are thus two important phases of data analysis. Sensitivity or robustness analysis can be performed by the inclusion of a new variable in the existing model by the exclusion of an employed variable from the model or by substituting an alternative indicator of the same variable [47].

Life expectancy data disaggregated by gender allows for a more nuanced analysis, and we incorporate this information in our sensitivity analysis. Table 3, Table 4 present the estimation outcomes for male and female life expectancy at birth (MLE and FLE) respectively. Various static and dynamic model specifications employed in these regressions demonstrate statistical validity at conventional significance levels and pass relevant post-estimation tests. Across all model specifications, our analysis reveals consistent findings: access to clean fuels and technologies, food deficit, per capita health expenditure, primary education, immunization, safe drinking water, improved sanitation facilities, and lag of life expectancy emerge as statistically significant determinants of both MLE and FLE. In addition, we explore undernourishment as an alternative measure of nutritional adequacy substituting the food deficiency variable. The results of this alternative estimation, presented in Table 5, Table 6, Table 7, mirror those in Table 2, Table 3, Table 4, respectively, in terms of variable signs and statistical significance. This robustness across different settings and specifications underscores the reliability and consistency of our findings, lending further credence to the identified determinants of life expectancy for both genders.

**Table 3:** Determinates of Life Expectancy of Males.

Life Expectancy of Males at Birth (MLE) is the dependent variable						
Model Variable	Pooled OLS	Fixed Effect	Random Effect		System GMM	Difference GMM
Access to Clean Fuels and Technologies (A2C)	0.025*** (0.002)	0.009*** (0.003)	0.011*** (0.004)		0.006*** (0.000)	0.006*** (0.002)
Food Deficit (Kcals per person/day)	−0.017*** (0.002)	−0.010*** (0.001)	−0.009*** (0.003)		−0.001*** (0.000)	−0.001*** (0.000)
Health Expenditure Per Capita	0.002** (0.001)	0.007*** (0.001)	0.006*** (0.001)		0.001*** (0.000)	0.001*** (0.000)
Primary Education Male	0.002** (0.001)	0.063*** (0.005)	0.028*** (0.004)		0.003*** (0.000)	0.002*** (0.000)
Immunization	0.077*** (0.010)	0.029*** (0.009)	0.043*** (0.007)		0.004*** (0.000)	0.002*** (0.000)
Safe Drinking Water	0.066*** (0.013)	0.222*** (0.013)	0.236*** (0.019)		0.023** (0.011)	0.002** (0.000)
Improved Sanitation	0.058*** (0.005)	0.052*** (0.009)	0.063*** (0.011)		0.008*** (0.003)	0.003** (0.001)
Male Life Expectancy_t-1_					0.958*** (0.002)	0.917*** (0.002)
Intercept	3.253*** (0.058)	1.932*** (0.056)	2.257*** (0.070)		0.184*** (0.010)	0.263*** (0.014)
N	1446	1446	1446		1349	1172
F / χ^2^ Statistic [p values]	552.419*** [0.000]	999.37*** [0.000]	1859.784*** [0.000]		534,556.1*** [0.000]	198,982.98*** [0.000]
R^2^	0.728	0.972			0.728	
ρ (rho)			0.927			
F test (Fixed Effect) [p values]		153.610*** [0.000]				
Breusch-Pagan LM χ^2^ test [p values]			6803.83*** [0.000]			
Hausman Test [p values]		357.56*** [0.000]				
AR (1) [p values]					0.045	0.004
AR (2) [p values]					0.111	0.115
Sargan [p values]					0.705	0.799

N.B.: ***, ** and * denotes significance of coefficients at level < 0.01, <0.05 and < 0.100 respectively.

() reports standard errors and [] reports *p* value.

**Table 4:** Determinates of Life Expectancy of Females.

Life Expectancy of Females at Birth (FLE) is the dependent variable						
Model Variable	Pooled OLS	Fixed Effect	Random Effect		System GMM	Difference GMM
Access to Clean Fuels and Technologies (A2C)	0.029*** (0.002)	0.011*** (0.003)	0.013*** (0.004)		0.006*** (0.000)	0.007*** (0.001)
Food Deficit (Kcals per person/day)	−0.007*** (0.002)	−0.009*** (0.002)	−0.008*** (0.003)		−0.001*** (0.000)	−0.001*** (0.000)
Health Expenditure Per Capita	0.004*** (0.001)	0.006*** (0.001)	0.006*** (0.001)		0.001*** (0.000)	0.003*** (0.000)
Primary Education Female	0.002** (0.001)	0.064*** (0.006)	0.028*** (0.004)		0.004*** (0.000)	0.003*** (0.001)
Immunization	0.078*** (0.009)	0.033*** (0.009)	0.047*** (0.008)		0.001*** (0.001)	0.002*** (0.000)
Safe Drinking Water	0.050*** (0.012)	0.207*** (0.015)	0.217*** (0.020)		0.002** (0.000)	0.003** (0.001)
Improved Sanitation	0.086*** (0.005)	0.057*** (0.008)	0.076*** (0.012)		0.008** (0.004)	0.010** (0.004)
Female Life Expectancy_t-1_					0.992*** (0.001)	0.933*** (0.002)
Intercept	3.215*** (0.054)	2.003*** (0.083)	2.324*** (0.074)		0.178*** (0.015)	0.161*** (0.014)
N	1446	1446	1446		1349	1172
F / χ^2^ Statistic [p values]	808.653*** [0.000]	1038.642*** [0.000]	1695.409*** [0.000]		409,128.1*** [0.000]	424,644.57*** [0.000]
R^2^	0.796	0.974				
ρ (rho)			0.932			
F test (Fixed Effect) [p values]		161.907*** [0.000]				
Breusch-Pagan LM χ^2^ test [p values]			6912.58*** [0.000]			
Hausman Test [p values]		160.17*** [0.000]				
AR (1) [p values]					0.004	0.005
AR (2) [p values]					0.170	0.175
Sargan [p values]					0.776	0.736

N.B.: ***, ** and * denotes significance of coefficients at level < 0.01, <0.05 and < 0.100 respectively.

() reports standard errors and [] reports *p* value.

**Table 5:** Determinates of Life Expectancy.

Life Expectancy at Birth (LE) is the dependent variable							
Model Variable	Pooled OLS	Fixed Effect	Random Effect		System GMM	Difference GMM	
Access to Clean Fuels and Technologies (A2C)	0.027***	0.007***	0.009***		0.003***	0.007***	
	(0.002)	(0.003)	(0.004)		(0.000)	(0.003)	
Undernourishment	−0.020***	−0.015***	−0.015***		−0.003***	−0.001***	
	(0.003)	(0.003)	(0.004)		(0.000)	(0.000)	
Health Expenditure Per Capita	0.003***	0.007***	0.006***		0.001***	0.001***	
	(0.001)	(0.001)	(0.001)		(0.000)	(0.000)	
Primary Education	0.003***	0.063***	0.027***		0.002***	0.003***	
	(0.001)	(0.005)	(0.004)		(0.000)	(0.000)	
Immunization	0.071***	0.031***	0.044***		0.005***	0.002***	
	(0.009)	(0.009)	(0.007)		(0.001)	(0.000)	
Safe Drinking Water	0.054***	0.209***	0.221***		0.021**	0.002**	
	(0.012)	(0.018)	(0.020)		(0.011)	(0.001)	
Improved Sanitation	0.072***	0.053***	0.070***		0.006***	0.003**	
	(0.005)	(0.009)	(0.011)		(0.000)	(0.001)	
Life Expectancy_t-1_					0.979***	0.925***	
					(0.003)	(0.002)	
Intercept	3.271***	2.012***	2.336***		0.107***	0.215***	
	(0.054)	(0.088)	(0.074)		(0.012)	(0.015)	
N	1435	1435	1435		1338	1162	
F / χ^2^ (Model) Statistic [p values]	712.179*** [0.000]	1343.832*** [0.000]	1808.178*** [0.000]		385,547.4*** [0.000]	461,163.3*** [0.000]	
R^2^	0.776	0.974					
ρ (rho)			0.929				
F test (Fixed Effect) [p values]		157.396*** [0.000]					
Breusch-Pagan LM χ^2^ test [p values]			6819.57*** [0.000]				
Hausman Test [p values]		142.37*** [0.000]					
AR (1) [p values] 0.001							0.002
AR (2) [p values] 0.100							0.101
Sargan [p values] 0.850							0.769

N.B.: ***, ** and * denotes significance of coefficients at level < 0.01, <0.05 and < 0.100 respectively.

() reports standard errors and [] reports p value.

**Table 6:** Determinates of Life Expectancy of Males.

Life Expectancy of Males at Birth (MLE) is the dependent variable							
Model Variable	Pooled OLS	Fixed Effect	Random Effect		System GMM	Difference GMM	
Access to Clean Fuels and Technologies (A2C)	0.024***	0.006**	0.008**		0.007***	0.008***	
	(0.002)	(0.003)	(0.004)		(0.003)	(0.002)	
Undernourishment	−0.027***	−0.015***	−0.016***		−0.001***	−0.001***	
	(0.003)	(0.003)	(0.004)		(0.000)	(0.000)	
Health Expenditure Per Capita	0.002*	0.007***	0.006***		0.007***	0.001***	
	(0.001)	(0.001)	(0.001)		(0.001)	(0.000)	
Primary Education Male	0.003***	0.062***	0.027***		0.063***	0.002***	
	(0.001)	(0.005)	(0.004)		(0.005)	(0.000)	
Immunization	0.072***	0.029***	0.041***		0.031***	0.002***	
	(0.010)	(0.008)	(0.007)		(0.009)	(0.000)	
Safe Drinking Water	0.059***	0.218***	0.231***		0.023**	0.002**	
	(0.013)	(0.016)	(0.020)		(0.011)	(0.001)	
Improved Sanitation	0.059***	0.051***	0.064***		0.008***	0.004**	
	(0.005)	(0.009)	(0.011)		(0.001)	(0.002)	
Male Life Expectancy_t-1_					0.960***	0.918***	
					(0.002)	(0.003)	
Intercept	3.289***	1.972***	2.299***		0.172***	0.245***	
	(0.058)	(0.073)	(0.073)		(0.011)	(0.015)	
N	1435	1435	1435		1338	1162	
F / χ^2^ (Model) Statistic [p values]	557.116 *** [0.000]	1712.688*** [0.000]	1851.963*** [0.000]		443,418.7*** [0.000]	233,592.63*** [0.000]	
R^2^	0.731	0.972					
ρ (rho)			0.927				
F test (Fixed Effect) [p values]		153.144*** [0.000]					
Breusch-Pagan LM χ^2^ test [p values]			6788.46*** [0.000]				
Hausman Test [p values]		137.39*** [0.000]					
AR (1) [p values] 0.041							0.047
AR (2) [p values] 0.111							0.115
Sargan [p values] 0.596							0.665

N.B.: ***, ** and * denotes significance of coefficients at level < 0.01, <0.05 and < 0.100 respectively.

() reports standard errors and [] reports p value.

**Table 7:** Determinates of Life Expectancy of Females.

Life Expectancy of Females at Birth (FLE) is the dependent variable							
Model Variable	Pooled OLS	Fixed Effect	Random Effect		System GMM	Difference GMM	
Access to Clean Fuels and Technologies (A2C)	0.028***	0.009***	0.011***		0.004***	0.006***	
	(0.002)	(0.004)	(0.004)		(0.000)	(0.002)	
Undernourishment	−0.014***	−0.015***	−0.015***		−0.003***	−0.001***	
	(0.003)	(0.003)	(0.004)		(0.000)	(0.000)	
Health Expenditure Per Capita	0.004***	0.007***	0.006***		0.001***	0.001***	
	(0.001)	(0.001)	(0.001)		(0.001)	(0.000)	
Primary Education Female	0.003***	0.064***	0.027***		0.002***	0.004***	
	(0.001)	(0.006)	(0.004)		(0.000)	(0.001)	
Immunization	0.074***	0.033***	0.046***		0.006***	0.002***	
	(0.009)	(0.009)	(0.008)		(0.001)	(0.000)	
Safe Drinking Water	0.044***	0.200***	0.210***		0.002**	0.003**	
	(0.012)	(0.019)	(0.021)		(0.001)	(0.001)	
Improved Sanitation	0.087***	0.056***	0.076***		0.020***	0.010**	
	(0.005)	(0.008)	(0.012)		(0.002)	(0.003)	
Female Life Expectancy_t-1_					0.992***	0.933***	
					(0.002)	(0.001)	
Intercept	3.254***	2.050***	2.373***		0.074***	0.163***	
	(0.054)	(0.104)	(0.076)		(0.012)	(0.015)	
N	1435	1435	1435		1338	1162	
F / χ^2^ (Model) Statistic [p values]	803.096*** [0.000]	1166.691*** [0.000]	1690.330*** [0.000]		385,411.6*** [0.000]	424,644.57*** [0.000]	
R^2^	0.797	0.975					
ρ (rho)			0.932				
F test (Fixed Effect) [p values]		161.624*** [0.000]					
Breusch-Pagan LM χ^2^ test [p values]			6895.31*** [0.000]				
Hausman Test [p values]		110.69*** [0.000]					
AR (1) [p values] 0.003						0.004	
AR (2) [p values] 0.124						0.176	
Sargan [p values] 0.884						0.735	

N.B.: ***, ** and * denotes significance of coefficients at level < 0.01, <0.05 and < 0.100 respectively.

() reports standard errors and [] reports p value.

The statistical significance of common determinants across various model specifications reinforces their importance in shaping life expectancy outcomes for both males and females. Access to clean fuels and technologies, along with factors such as nutrition, healthcare expenditure, education, and sanitation, emerges as critical drivers of population health. These findings underscore the multidimensional nature of health outcomes and highlight the interconnectedness of socioeconomic, environmental, and healthcare factors in influencing life expectancy. As such, policymakers should prioritize interventions that address these determinants comprehensively to improve population health and enhance overall well-being. Moreover, the robustness of our results under different analytical approaches strengthens the evidence base for informed policy decision-making. By demonstrating the consistency of findings across various estimation techniques, our study provides policymakers with confidence in the identified determinants of life expectancy and underscores the need for targeted interventions addressing these factors. Moving forward, policies aimed at improving access to clean energy, enhancing nutritional sufficiency, expanding healthcare services, and promoting education and sanitation can play pivotal roles in fostering population health improvements and achieving sustainable development goals.

## 5 Conclusion

Development is the process involving the improvement of population health. Therefore, one of the principal goals of public policies today is to improve the quality of health by lengthening the life expectancy. The study's significance lies in its exploration of the often-overlooked role of clean fuel and technologies in enhancing life expectancy, a critical aspect of development and public health policy. While economists have extensively analyzed various factors influencing life expectancy, such as healthcare expenditure, education, and sanitation, the specific impact of access to clean fuels has been relatively neglected in empirical research. By addressing this gap, the study brings attention to an essential yet underexamined determinant of population health, shedding light on the potential benefits of investing in clean energy solutions. Furthermore, the use of rigorous panel data analysis techniques adds credibility to the study's findings and enhances its policy relevance. By analyzing data from 190 countries over two decades drawn from the World Bank [15], the study provides a comprehensive and robust assessment of the relationship between clean fuel access and life expectancy controlling for unobserved heterogeneity, endogeneity, and autocorrelation. This methodological rigor strengthens the study's contribution to the existing literature and enhances its potential impact on policy formulation and implementation.

Overall, the study's findings carry significant implications for policymakers, highlighting the importance of prioritizing investments in clean fuels and technologies to improve population health and longevity. By demonstrating the positive association between access to clean energy and life expectancy while controlling for various confounding factors, the study provides empirical evidence to support the adoption of clean energy policies on a global scale. By acknowledging clean fuels as a determinant of life expectancy, policymakers can prioritize investments in clean energy infrastructure and technologies as part of broader development strategies. This shift in policy focus towards clean energy not only benefits public health but also contributes to environmental sustainability and economic development, creating a win-win scenario for communities and nations globally. Finally, the study's findings have significant implications for reshaping public policies in the clean energy-poor countries in the Global South. Domestic and international policy interventions aimed at alleviating clean energy poverty and scaling up access to clean fuels and technologies are required to enhance both the duration and quality of life, thus fostering sustainable development efforts at both national and global levels. -By integrating clean energy policies into broader development strategies, policymakers can create synergies between economic growth, climate protection, and public health improvement, leading to more sustainable and resilient societies. Ultimately, the adoption of evidence-based policies informed by the research findings can contribute to achieving the overarching objectives of promoting human well-being and advancing global development goals.

While the study offers valuable insights into the relationship between clean fuel access and life expectancy, several limitations should be acknowledged. Firstly, the study relies on secondary data sources, such as those from the World Bank, which may be subject to measurement error, reporting biases, and inconsistencies across countries. Additionally, the study's reliance on aggregated national-level data may overlook variations in clean fuel access and health outcomes within countries, potentially masking important heterogeneity across regions, socioeconomic groups, and urban-rural divides. Future research could benefit from incorporating more granular, disaggregated data to better capture these local nuances and improve the generalizability of the findings. Furthermore, while the study employs advanced panel data analysis techniques to account for various sources of bias and endogeneity, certain methodological limitations remain. For instance, the study's focus on observational data precludes establishing causality between clean fuel access and life expectancy, as other unobserved factors may confound the relationship. Although efforts are made to control for potential confounders, such as healthcare expenditure and education, residual confounding and omitted variable bias could still affect the estimated effects. Additionally, the study's reliance on cross-country comparisons may overlook contextual factors and policy differences that could influence the observed associations. Future research could benefit from complementing quantitative analyses with qualitative studies and experimental designs to better elucidate the causal pathways and mechanisms underlying the relationship between clean fuel access and population health outcomes. More research could focus on rural-urban or regional or household-level inequality in clean energy access and their impacts on health outcomes.

## CRediT authorship contribution statement

**Amit Roy:** Writing – review & editing, Writing – original draft, Visualization, Validation, Supervision, Software, Methodology, Investigation, Formal analysis, Data curation, Conceptualization.

## Declaration of competing interest

The authors declare that they have no known competing financial interests or personal relationships that could have appeared to influence the work reported in this paper.
